# Individual experiences following a 6-month exercise intervention: A qualitative study

**DOI:** 10.3402/qhw.v10.26376

**Published:** 2015-08-14

**Authors:** Ellen Staveborg Kerkelä, Linus Jonsson, Magnus Lindwall, Jennifer Strand

**Affiliations:** 1 Department of Psychology, University of Gothenburg, Gothenburg, Sweden; 2 School of Health and Welfare, Halmstad University, Halmstad, Sweden; 3 Department of Food and Nutrition, and Sport Science, University of Gothenburg, Gothenburg, Sweden

**Keywords:** Self-determination theory, inductive approach, thematic analysis, motivational interviewing, dropout, adherence

## Abstract

**Purpose:**

Dropout is a common problem in various exercise interventions. The individual's experience is believed to greatly impact dropout, yet little is known about the individual experiences of taking part in exercise interventions. The aim of this study was to examine individuals’ experiences following a self-determination theory–based exercise intervention in order to gain understanding of how standardized interventions can be adjusted to fit individuals’ specific needs, capacities, and circumstances.

**Methods:**

A qualitative approach with semi-structured interviews was conducted with eight informants (three male and five female) aged between 26 and 47 years, whom all had participated in a 6-month exercise intervention with individual coaching based on self-determination theory and motivational interviewing. The interviews were analyzed thematically with an inductive approach.

**Results:**

Aspects that influenced the informants’ motivation and participation in the exercise intervention were linked to three themes: the frames of the intervention, measurable changes, and the individual's context. The themes present information about the process and to what extent the informants felt that the intervention was adapted to fit their lives and needs.

**Conclusions:**

This study emphasizes the importance of individualizing exercise interventions to support individuals’ diverse capacities and psychological needs.

The relationship between the physical body and the mind, as well as its influence on individual's health, has been discussed for centuries (Damasio, 1994), and at present there is consensus that somatic and mental health in various ways interact. Health-promoting research and recommendations from authorities in society suggest beneficial effects of physical activity (PA) and exercise on individuals’ physical and mental health (Penedo & Dahn, 2005; Warburton, Nicol, & Bredin, 2006). This applies both from a preventive, as well as from a treatment perspective, which is indicated by the fact that PA is a recurring element in psychological treatment of, for example, depression, anxiety, stress, and sleep disorders (Josefsson, Lindwall, & Archer, 2014; Lindwall, Gerber, Jonsdottir, Börjesson, & Ahlborg, 2013; Westbrook, Kennerley, & Kirk, 2011).

In accordance with evidence-based practice, there is an increasing request to use efficient, concise, and cost-effective treatments, that includes a holistic perspective (APA, 2006; Haase, Taylor, Fox, Thorp, & Lewis, 2010; WHO, 2008). Consequently, an important aspect of facilitating public health and reducing health-related costs is to promote long-term behavior changes with lasting health benefits. Thus, increasing individuals’ PA levels is viewed as a central health-promoting strategy. However, many individuals experience difficulties initiating and maintaining a regular exercise regimen (Ng et al., 2012). In order to experience the positive health benefits associated with PA, it is often required to stay active for 6 months or more, resulting in many individuals dropping out before the positive effects emerges (White, Ransdell, Vener, & Flohr, 2005). In light of this, it is essential to examine potential developments for interventions so they become more accessible and adapted to individuals’ specific needs, circumstances, and capacities.

The significance of changing and creating permanence in behavior has resulted in growing research within the field of motivation in order to examine factors promoting, initiating, optimizing, and thwarting individuals’ motivation (Deci & Ryan, 2000; Vansteenkiste, Williams, & Resnicow, 2012). One theory of motivation that has gained increasing support in the area of PA and exercise is self-determination theory (SDT; Deci & Ryan, 2000; Ryan & Deci, 2000; see Teixeira, Carraça, Markland, Silva, & Ryan, 2012 for a review).

## Self-determination theory

One basic assumptions of SDT is that all humans have a natural tendency and desire to be active organisms and to control their lives in a desired direction (Deci & Ryan, 2000; Ryan & Deci, 2000). This assumption, of a natural movement, includes the wish to move toward increased well-being, growth, maturation, and an integrated self. Within SDT, the individual is viewed as in constant interaction with its context. This interaction affects the natural tendency toward development by stimulating or thwarting satisfaction of the three basic psychological needs; autonomy, competence, and relatedness. Satisfaction of the need for autonomy results in a sense of psychological freedom and choice. The need for competence is the ability to believe in oneself, feeling capable, and that one can manage what one might face. Relatedness includes the need to feel confident in a particular environment, being able to experience intimacy and support, and having meaningful relationships (Deci & Ryan, 2000; Hagger & Chatzisarantis, 2008).

SDT stipulates that development and persistency of a behavior and behavior change is dependent on the quality of the motivation (Deci & Ryan, 2000). On the one hand, when an individual is driven by “low quality” motivation (i.e., amotivation, external, and introjected regulation), the behavior is perceived as controlled and driven by external factors. One the other hand, when an individual is driven by “good quality” motivation (i.e., identified, integrated, and intrinsic regulation), the behavior is more self-determined and internalized (see Deci & Ryan, 2000; Ryan & Deci, 2000). Furthermore, according to SDT, satisfaction of the psychological needs increases the quality of motivation (Deci & Ryan, 2000; Ryan & Deci, 2000).

The movement toward becoming more self-determined involves external norms, values, and regulations being internalized, resulting in integration with important parts of the self and central beliefs of the identity. This creates a solid foundation for maintaining a certain behavior (Patrick & Williams, 2012). Primarily the autonomous forms of motivation (identified, integrated, and intrinsic regulation) have been positively associated with maintenance of PA and exercise (Ng et al., 2012; Silva et al., 2010; Teixeira et al., 2012).

## Qualitative approach and self-determination theory

The tradition and ideal to establish knowledge by randomized controlled trials (RCTs) has been challenged in the psychological and social domains in order to gain legitimacy for a variety of methods in producing knowledge and evidence (APA, 2006; Biddle, Markland, Gilbourne, Chatzisarantis, & Sparkes, 2001; Bohlin & Sager, 2011). As such, there has been an increased call for qualitative studies, as they can increase understanding for aspects that contribute to or limit the effectiveness of exercise interventions. Yet few studies have been conducted that have explored individual experiences from taking part in different exercise intervention (e.g., Huberty et al., 2013; Moore, Moore, & Murphy, 2011; O'Sullivan et al., 2010). The qualitative studies that exist have, however, highlighted factors related to engagement, participation, and to some extent, long-term behavior change.

### The instructors’ importance and approach

The instructors’ enthusiasm, interest, and encouragement, as well as knowledge and ability to give practical advice, are some factors emphasized for participation in exercise interventions. These qualities were described to help the participants to develop new skills, techniques, and strategies to change habits and to overcome barriers (Moore et al., 2011; O'Sullivan et al., 2010; Podlog & Dionigi, 2009). Huberty et al. (2013) found that support to develop self-regulation strategies, such as goal-setting, increased persistence, and those participants who used goals continued to exercise to a greater extent. Personal, realistic, and challenging goals were considered useful to get feedback on improvements. In the study by Podlog and Dionigi (2009), the instructors’ support was critical in order for the participants to start using their bodies, regain physical abilities, and acquire new skills, resulting in feelings of satisfaction and achievement. O'Sullivan et al. (2010) further established that the instructors’ characteristics influenced the participants’ perception of their relationship, which in turn affected their motivation. An autonomy supportive instructor (autonomy support refers to what a person says and does to enhance another's internal perceived locus of causality, volition, and perceived choice; Reeve, Nix, & Hamm, 2003) appeared vital to increase motivation and feelings of competence (Moore et al., 2011; O'Sullivan et al., 2010; Podlog & Dionigi, 2009), and freedom of choice was considered important for long-term exercise (Huberty et al., 2013).

### Social support

The development of social networks has been found vital to create long-term behavior change (Moore et al., 2011; Podlog & Dionigi, 2009). In the study by Podlog and Dionigi (2009), the participants described friendship and camaraderie as crucial for their engagement and participation in the exercise intervention. Both the importance of concrete support, such as being able to push and help each other, as well as emotional support was emphasized. The meaning of social networks was also demonstrated by feelings of obligation in relation to other group members and the family (Podlog & Dionigi, 2009). Whereas the commitment to the group was described as a positive aspect, perceived obligations to the family had a negative influence on the individual's participation. Difficulties in balancing and prioritizing among important factors in life and lack of support from the family have been found to arouse feelings of guilt and duty, often resulting in decreased adherence and dropout from interventions (Huberty et al., 2013; Podlog & Dionigi, 2009).

### Exercise environment and role models

As many beginners were insecure about the exercise environment, the instructors in the study by Moore et al. (2011) reflected upon the importance of reducing anxiety related to the exercise setting for participants to stay motivated. Furthermore, environments that offer varying forms of exercise were described as more enjoyable and increased feelings of choice. In addition, these environments offered greater potential to develop new abilities (Huberty et al., 2013; Podlog & Dionigi, 2009). Also, being able to exercise in places where focus is not on appearances or showing off has been empathized (Huberty et al., 2013; Moore et al., 2011; O'Sullivan et al., 2010; Podlog & Dionigi, 2009) and an empathetic and socially permissive environment has been considered to increase social support and offer more realistic role models (Moore et al., 2011). The importance of aforesaid was stressed by Huberty et al. (2013) who found that individuals who continued to exercise on a long-term basis were more accepting and had more positive views of their bodies.

## The present study: purpose

It is common that individuals experience difficulties maintaining a regular exercise routine (Ng et al., 2012) and that they for various reasons drop out (White et al., 2005). Therefore, it is essential to examine factors that influence dropout and long-term behavior change. The tradition of evidence has strongly focused on RCTs, but for psychosocial interventions there is a need to open up for qualitative studies to broaden the knowledge base (Biddle et al., 2001; Bohlin & Sager, 2011). By using a qualitative approach, this research intends to examine individuals’ experiences following an SDT-based exercise intervention in order to gain understanding of how standardized interventions can be adjusted to fit individuals’ specific needs, capacities, and circumstances.

## Method

### Intervention

The present study had its origin in an exercise intervention designed within an ongoing stress-related study in Gothenburg. The aim was to investigate the impact of PA on stress reactions and stress-related illness. The study is a registered trial at www.clinictrails.gov (#NCT02051127) and has been approved by the regional ethical committee (#917-12). By advertisement in local newspapers, social media, and posters, 100 individuals were recruited and randomized to an active or a control group. To be included, the individuals had to be physically active (i.e., not report more than 2 h of light PA per week); further they had to report at least 50% full-time occupational activity or study tempo. Exclusion criteria were signs of illness such as diabetes or asthma, and medical use that could affect outcome variables. Persons with a body mass index (BMI) at 35 or above, or 18.5 or less were also excluded.

Initially all participants were tested to measure their health and stress levels. One test was performed on an exercise bike to estimate oxygen uptake, and another test was a psychological stress test. After performing the tests, blood samples were taken and indicators of stress levels were measured. The same tests were repeated at the end of participation. After this first stage of the intervention, the active group was given free access to a specific gym, a pulse-watch, and an internet-based diary, whereas the control group continued their lives as before. The active group agreed to perform condition-based exercise, holding an average pulse at 75% of estimated maximum pulse, about 1 h, three times per week for a period of 6 months. Furthermore, the active group had a coach whom by oral coaching aimed to increase the participants’ motivation to stay physically active. The coaching took place about every fifth week and began with an initial meeting, followed by two shorter telephone follow-ups, and a final concluding meeting. The coach had no contact with the participants between these coaching sessions.

The coaching was based on the SDTs main principles, that is, the provision of autonomy support, structure (i.e., the extent to which a social content is structured, predictable, contingent, and consistent), and interpersonal involvement (i.e., expression of affection, warmth, care, and nurturance; Markland, Ryan, Tobin, & Rollnick, 2005; Skinner & Edge, 2002; Tessier, Sarrazin, & Ntoumanis, 2010). It also included motivational interviewing (MI)-techniques such as taking an empathic stance, helping to clarify and set realistic goals, helping to develop problem solving and planning, and foreseeing barriers (Markland et al., 2005). SDT served as a guiding framework for the coaching, and the coaching was practiced through borrowing techniques of MI. The integration of SDT and MI has been advocated previously, and it aims to extend the existing knowledge base and the development of interventions to promote health behaviors (Patrick & Williams, 2012; Resnicow & McMaster, 2012; Vansteenkiste et al., 2012). It has been argued that MI lacks theoretical underpinnings, which makes it difficult to understand why a behavior change may occur or not. Furthermore, SDT lacks the tools to work with behavioral change in practice. As such, a potential integration provides both a guiding framework (i.e., SDT) and the necessary practical tools (i.e., MI) for behavioral change work (Patrick & Williams, 2012; Resnicow & McMaster, 2012; Vansteenkiste et al., 2012).

### Informants

The informants were eight individuals who had finished the intervention. Five informants were women and three were men. The age span was 26–47 years. Five informants had children and lived with a partner, and of the remaining, two lived with a partner and one was single. Two informants were studying at university, and the remaining six were employed. Of the six employed, five reported long educations at university. All informants had started and finished their involvement in the intervention at different times. The informants varied in how long they had been physically inactive before they took part in the intervention. Some said that they had always been inactive, whereas others spoke of 1–2 years inactivity. During the participation, three of the eight informants had stopped exercising for various reasons and did not repeat the bicycle and stress test at the end of the intervention.

### Procedure and interview

In total, ten individuals initiated their participation in the intervention, but one dropped out at an early phase and did not match the target group. The remaining nine individuals were contacted and eight agreed to participate in an interview. The ninth person declined to be interviewed due to lack of time. Some of the informants were initially invited to participate in the present study by the coach they had met during the intervention. The coach informed them about the study and that the first author would stay in touch. The remaining informants were directly contacted by the first author who provided information about what participation would include. The informants were also given information about confidentiality, that the interview would be recorded, and how the results would be presented.

The interviews were held in a separate room that is regularly used for clinical practice, at the Department of Psychology. The context was chosen because the first author at the time of writing studied at the premises. Before the interviews were carried out, the informants were given information about the focus of the study, the procedure of the interview, how data would be used, volition to answer questions, the right to stop the interview without giving a reason, and confidentiality. After consent, all informants were interviewed by the first author using a semi-structured interview guide designed for the present study. As such, the procedure followed the American Psychological Association's ethics code of conduct in research (cf. Sales & Folkman, 2000). Topics that the interviews focused on were the informants’ exercise background; the decision to participate in the intervention including goals and expectations; and physical, psychological, and social aspects that they considered extra helpful, lacking, and/or demanding. Furthermore, the informants were asked about internal and external changes; benefits and losses they had experienced socially, psychologically, and physically; and their future outlook on exercise. The duration of the interviews ranged from 1 to 1½ h, and all interviews were audio recorded. All questions were open-ended, and follow-up questions were used in order to gain deeper understanding of areas that seemed essential for each individual. All interviews were transcribed by the first author.

### Analysis

Thematic analysis was chosen to analyze data as it is a flexible method that allows themes to emerge from data. Furthermore, the thematic analysis is not derived from any specific theoretic stance or epistemological position (Braun & Clarke, 2006), which is in line with the purpose of the present study. Due to the explorative nature of the study, an inductive approach was chosen. Our intention was to be open and, as far as possible, step aside from preconceptions and pre-understandings. In the present case, it should be noted that the first author, whom performed the analysis, was familiar with the literature of the focus area, and also had studied psychology for about 5 years.

The data were analyzed using a thematic analysis, following the recommendations of Braun and Clarke (2006). Initially, the first author read and reread all interview transcripts to obtain a sense of the whole and to become familiar with the data. During the second reading, an initial coding was conducted and reflections were noted in one of the margins. At this early stage of the analysis, it was common that one passage had more than one code. During the second reading, the transcripts were recoded and the number of codes was reduced to a final code. When about it was unclear what code that was most suitable, this was discussed among the authors in the research group.

Subsequently, all transcripts were cut into pieces to make it easier to see the codes as separate units and to be able to work with the material in physical form. The coded units were reread and collected in sheaves that came to represent provisional subthemes. The provisional subthemes were reread in order to find structure, and as this proceeded, some subthemes were joined together and created the three main themes. To satisfy Elliott, Fischer, and Rennie's (1999) criterion for credibility checks, the possible structure of themes and subthemes was continuously discussed in the research group. When differences arose, these were discussed and agreement was reached about the most suitable interpretation. Finally, the coded data extracts were reread to ensure that they covered the aim of the study. The data extracts were reviewed to find examples that best captured the participants’ experiences.

## Results

The thematic analysis resulted in three main themes and 10 subthemes related to the informants’ adherence, motivation, and participation. The themes are described below and illustrated by quotes that are characteristic for each theme. Fictitious names are being used. Themes and subthemes are presented in Table I.

**Table I:** Themes and subthemes.

Main theme	Sub-theme
1. The frames of the	1.1 The structure as encouraging
intervention	1.2 The structure as restraint
	1.3 The relationship to the coach
	1.4 Individualized exercise
2. Measurable changes	2.1 To notice change
	2.2 Increased self-awareness
	2.3 The assimilation process
3. The individuals’	3.1 Effects on everyday life
context	3.2 Social support
	3.3 Difficulties prioritizing

### The frames of the intervention

The theme revolves around aspects of the structure and agreement of participating, including the meaning and expectations that the informants related to partaking in the intervention.

#### The structure as encouraging

Once accepted to take part in the intervention, several informants’ experienced a positive change in their attitude toward exercise. To take responsibility for oneself, as well as to participate and have someone's approval to exercise, was mentioned to contribute to the positive changes. The fact that the aim of the study was considered important increased the informants’ willingness to complete the agreement and deliver good results.

Speaking of the cognitive and physiological test, it became really clear that there was a huge potential for improvements. I thought it was a well-executed and trustworthy study. (Edvin)

The tests that the informants took at the beginning of their participation were considered as motivational as they measured their physical capacity and ability to handle stress. By doing so the potential for change was clarified. The detailed information about intensity, amount, and types of exercise was described as helpful and had encouraged the informants’ when they felt exhausted, ill, or unmotivated.

If you have been sick for a week it might take a long time to get back but being a part of the study I found it easier to get back out because I felt, well I'm better now so I'd better get myself out. (Filippa)

#### The structure as restraint

The initial stress test was perceived as a demanding task according to all informants. The direct impact on the exercise performance was generally not outspoken, but some informants spoke of states of agitation and feelings of failure.

It was that feeling, the feeling afterwards that I was useless and then, the information. I would have liked to be informed that, you know, everyone went through the same; that it was this way or that way to all of us. (Gabriel)

To some informants, it got to a point where they said that they would warn others about the stress test. Others mentioned that thinking about the stress test, combined with stressful life circumstances, made them skip the final tests. Moreover, the informants spoke ambivalently about the exercise request and many considered their loyalty and initial motivational effects of the exercise agreement to become stressful.

The only thing that affected me negatively was the demands I put on myself, I felt so loyal and that I let them down, that I did not deliver what was expected of me, and really, that's just silly because it is a voluntary study. (Agnes)

The stress resulted in increased guilt, anxiety, and feelings of failure. The devotion and obligation made some informants’ reflect upon the aim of the study and the experts’ responsibilities, including their capacity to meet the individuals’ specific needs.

The whole time I was thinking that I have a responsibility, being accountable and producing results for the study. And I was thinking, if I didn't have [name] things might not have gone so well. (Dainella)

#### The relationship to the coach

Being able to access a coach was considered important and appealing when the informants applied to the study, and several informants’ spoke positively about the coach's sensible and accepting approach.

They have stayed in touch so you haven't been left on your own but they have not been too pushy or annoying. I find it annoying if someone is on my back the whole time, they have been a great support. (Agnes)

To get support and affirmation, and that the effort was considered important to someone else, was mentioned as crucial. The coach helped the informants’ to establish and concretize their goals and encouraged them by getting advice, strategies, and ideas about how to improve their exercise. However, some informants had ambivalent feelings about the coach and spoke of a lack of positive input and that they would have preferred more continuous contact.

A coach will not only help during those [coaching] occasions, but will keep a watchful eye the whole time to help correct eventual slips on the way. And yeah there was someone to point out the direction but no one to let you know when you left it. (Edvin)

Others experienced the contact as deficient, resulting in disappointment and questioning of the coach's ability to adjust to the individuals’ need for support.

I thought I was going to get a program tailored after me. That I was going to have a coach to talk about my needs with and not the needs of the study and perhaps having some kind of dialogue, but that never happened. We went through a series of question that the coach had and that didn't give me anything. (Cecilia)

Variation also became evident as some described that they would have preferred a coach that was present in the exercise environment, whereas others expressed the opposite.

#### Individualized exercise

To avoid obligation and stress associated with achieving and delivering results, the informants described it as central that the exercise was fun and easy. It was also crucial to make the exercise fit in with their everyday routines. To be informed and have knowledge about PA was emphasized, in order to establish workout routines that were indulgent and realistic.

I guess I had a different way of approaching it when I got into it this time, put up a reasonable level instead of peaking, maybe not peak, but jump right into it and push myself to the limits, because if I do that I know it will end up with the motivation dropping and injuries starting to appear. (Henry)

The possibility to individualize the exercise routines was influenced by the agreements in the intervention. Specifics on the amount, intensity, and type of exercise were sometimes considered as stressful and/or restricting, and strategies were developed to manage what was expected.

I found my own strategy, switched and took turns between four different machines at the gym to get those 45 min of high intensive exercise done, but it was never helpful and perhaps I wanted to lift some weights, but didn't know how to, I wasn't sure if I did it right or wrong. (Cecilia)

To be referred to a gym was by some informants experienced as uncomfortable as they found the gym environment impersonal or described feeling unaccustomed at the gym. To book gym classes and long travelling distances were other aspects considered to complicate and reduce joy and possibilities to exercise.

### Measurable changes

The theme concerns the changes that the informants experienced during the intervention. It involves the actual measurable changes in physic and achievements, and the more diffuse changes that they experienced within themselves.

#### To notice change

The informants emphasized the meaning of concrete and noticeable improvements. More obvious changes described were improved posture and sleep, increased strength and muscles. The exercise was further described to increase satisfaction and energy in some informants’ everyday lives.

I had energy and I didn't find it so hard to do the everyday chores, I got them done and felt more pleased with myself because the home was in better order and I could go to bed with both a better conscious and feeling tired in a good way. (Edvin)

Many informants spoke of increased curiosity and joy using the pulse-watch and exercise diary as it helped them keep track and compete with earlier performances. The equipment moderated self-criticism and helped the informants to recognize and understand their physical capacity and signals, something that many had found troublesome earlier in life.

I haven't had any knowledge and then I haven't had any motivation so I have given up after 2 weeks, because how will I know when it gives results, I haven't been able to know. (Daniella)

To get detailed information from each workout helped the informants to appreciate their achievements and realize that an exercise routine had been established.

#### Increased self-awareness

There was a large variety among the informants’ approach and use of goals during the intervention. Commonly the informants spoke of initial difficulties to put up reasonable and realistic goals, but experienced it easier as their self-awareness increased during the intervention.

Trying to set up goals was incredible hard as you don't know your own level, you don't know what you might be capable of in 3 months with that kind of exercise. I think I was just lacking knowledge because I didn't know my level at the start. (Gabriel)

Realistic and concrete goals were considered crucial as it helped the informants’ to distinguish improvements. Furthermore, the goals created a structure and pointed out a direction, which was particularly useful after setbacks or disruptions in the exercise routine. Some informants used gradual goals as a strategy to prevent feelings of failure.

After some time I lowered my goals. Before I have been like “all-or-nothing” and let's say, if I return after a long break and cannot run 5 km in a certain pace I might feel really bad and think “nooo I can't do this.” But it is better to get out and just do a lap up and down the hill, and that's good enough. I think it's easier for me to think like that today, to have more … I don't know if they are realistic, but smaller goals. (Filippa)

#### The assimilation process

To some extent, all informants described a process where intellectual knowledge about the effects of exercise was assimilated into experiential knowledge. During this process, they experienced that they began to exercise to please themselves and that abstinence appeared when they did not exercise. The importance to feel abstinence was mentioned by informants who had stopped exercising during the intervention.

Since I never got to that … over to the other side of the hump where it feels good to exercise, instead I kept struggling, yeah I never got to that but if I would have, it would have been yet another motivational factor, a bell ringing letting me know that I am keeping up but no, I never got over to that side. (Henry)

Absence of results and not reaching a state where the exercise felt natural and easily performed led to disappointments and contributed to lack of motivation. The assimilation process involved changed expectations and new ways of thinking. Some mentioned increased guilt, and others experienced gradually becoming more permissive and trusting their ability to manage barriers. Most informants questioned, revised, and changed assumptions about their self-image.

I have left my very obstinate way of thinking, that exercise is not my thing. Today, I can see myself put the runners on and actually go out for a run, and I don't think I have been able to imagine that before, so some things must have changed in my mind. (Agnes)

### The individual's context

This theme includes the changes that the exercise had on the informants’ everyday lives and contextual aspects that influenced their participation. Social and relational aspects and its impact on choice and priorities are emphasized.

#### Effects on everyday life

The involvement in the intervention had impact on the informants’ lives. Some described that stress caused by participating had negative influences, but most informants experienced that increased energy, strength, and more permissive ways of thinking made it easier to handle barriers in their lives.

I think my approach to things is a bit more relaxed these days, I don't get as worked up or stressed about small things as I used to, it's easier to leave and let go of things. (Beatrice)

Planning was a factor that most informants considered crucial to make exercise a natural part of life. To some, it caused conflict as it meant denying something else. The relation between planning and motivation was illustrated when the routine had been disrupted.

It was hard during the summer with holidays and all that, when the everyday habits were disturbed, and I think that if I had kept the exercise routine throughout the holidays I might have found exercise easier during the autumn. (Edvin)

#### Social support

To have a partner or other close relationships to support, participate in, or motivate the new habits and exercise routines was important. The need for social support was further described in the exercise environment, and resulted in some informants experiencing a growth in their social lives. The importance of being noticed and attached to others was also depicted in the wish to have a more present coach, alternatively to exercise with a friend or to exercise in a group.

If it was more like a group thing it would, we would push each other and it would be easier to get it done. I mean, in that case you would get organized, compared to if you make all the decisions on your own, which would be different. (Gabriel)

Exercise in a group context was described as making it easier to prioritize exercise, and to facilitate challenge and competition. To be able to compare one to others mitigated feelings of being inferior or alone.

I was looking for someone who was in the same poor state as me, there is no competition with an individual like that but it was good to see that there were others who were not top-trained, yeah it was really good to see that I'm not the only one who has such a bad shape, it helped. (Henry)

#### Difficulties prioritizing

When the informants spoke of their exercise background, reasons that they had quit exercising became evident. Many described the distribution of responsibilities and duties in their private lives, and in their professional situation as vital for their ability to prioritize exercise.

I mean, if it wasn't for him [my husband] getting that promotion working so many late hours, I think I would have managed three workouts a week. (Agnes)

Many informants described that they tended to prioritize what they considered best for their family. Difficulties in distinguishing positive long-term consequences of exercise made it easy to justify short-term efforts and duties in everyday life, and some informants’ described that this pattern, plus a lack of energy, easily developed into a “lazy spiral.”

When I had been inactive for such a long time it was easier to justify other efforts, other things became much more important than to exercise. Exercising became such an exhausting thing to do. (Beatrice)

## Discussion

A growing body of research supports the positive effects of PA and exercise on physical and psychological well-being (e.g., Josefsson et al., 2014; Lindwall et al., 2013; Ng et al., 2012). To maintain individuals’ health development, the changes in health behaviors need to stay permanent and currently there are difficulties in promoting long-lasting changes, as exemplified by high dropout rates in exercise interventions (White et al., 2005; Williams, Hendry, France, Lewis, & Wilkinson, 2007). To gain understanding for how standardized interventions can be adjusted to fit individuals’ specific needs, capacities, and circumstances, the present study investigated individuals’ experiences of partaking in an exercise intervention. Aspects considered crucial to the informants’ experiences and participation in the intervention were related to three main themes: “the frames of the intervention,” “measurable changes,” and “the individual's context.”

O'Sullivan et al. (2010) discuss that exercise at the beginning generally is more exhausting than fun to perform and consequently needs determination to be retained. The importance of initial motivation is emphasized in contexts where the PA has a health promotive and/or preventive aim, as problems and symptoms may be absent to the individual, also making it hard to notice the effects of the exercise (O'Sullivan et al., 2010). The result in the present study underlines the importance of physical and psychological measurable changes and improvements for individuals that have been physically inactive, to maintain motivation and prioritize exercise and health behaviors on long-term basis. Initially, loyalty and the frames of the intervention were sufficient for the informants’ motivation. But eventually, it got hard for some informants to obtain and prioritize the exercise. The initial motivational effect due to the frames became a restraint for some informants, which contributed to stress, anxieties, feelings of guilt and failure, and sometimes dropout. Emotions, thoughts, and behaviors are closely intertwined, and associated with core beliefs about the self, in individuals’ minds (Westbrook et al., 2011), partly explaining how feeling pressured to achieve and deliver results, including emotions such as guilt and failure, can affect feelings of competence, motivation, and the participation negatively. It also suggests how thoughts and emotions regarding the stress test might have impacted the exercise routines negatively. In line with above mentioned, research has found that guilt has thwarting effects on exercise (Huberty et al., 2013) and that guilt, as well as feeling pressured, is associated with controlled forms of motivation that has negative consequences on exercise routines (Teixeira et al., 2012).

The initial doubt about the own capacity made many informants appreciate the pulse-watch to explore their bodies, and using the diary and goals helped them by pointing out a direction for progress. In accordance with earlier research (Huberty et al., 2013), the importance of realistic, personal, and tangible goals was empathized. The instruments provided “proof” of improvements and being able to notice change encouraged the informants to continue their PA, which gradually resulted in increased self-awareness, better coping, and being more permissive in their ways of thinking. Hence, attributes connected to autonomous motivation (cf. Hagger & Chatzisarantis, 2007) were accompanied with less negative emotions, which helped the informants to stay motivated, and facilitated long-term behavior change. The assimilation process resulted in positive emotions, and states of well-being were associated with PA and experiencing changes in the self-image. The evolving self-image was often described as including new values and a new direction that the informants tried to follow, aspects that are in line with increased autonomous motivation (Deci & Ryan, 2000).

The outcome in exercise interventions range depended on the intensity and kind of support offered (Duda et al., 2014), and the quality of the relationship with the coach has shown to affect motivation (O'Sullivan et al., 2010). Results in the present study underline the interplay between relational expectations and needs, motivation, and participation in interventions. Although some informants were satisfied with the form and intensity, others would have preferred the support to be more active, guiding, and continuous. Moore et al. (2011) found that diverse levels of anxiety associated with the exercise environment were reduced by individualized support. It suggests an explanation to the informants’ various needs of support during the intervention, as many mentioned insecurity about themselves and their capacity, and experienced discomfort at the gym. The expressions of anxiety may also be viewed from a broader self-presentational perspective on exercise (cf. Martin Ginis, Lindwall, & Prapavessis, 2007). Doubts in competence and capacity to successfully present oneself in a favorable way to others, in the particular context of the gym, may create negative effects and avoidance. In light of SDT, individuals differ in their satisfaction of psychological needs (Deci & Ryan, 2000) and when expectations about the coach's role and functioning were not met, it resulted in internal barriers such as disappointments. As a result some informants sought less practical or emotional support. Consequently, the coach may have failed to provide the informants with individualized support and structure, and thwarted their psychological needs. As highlighted by Jang, Reeve, and Deci (2010); it is not autonomy support, or structure; but autonomy support, and structure.

Beside the need for individualized support and structure, individualized exercise routines were crucial for maintaining motivation and participation. However, the possibility to individualize the PA was restricted by the detailed information about intensity, duration, and kind of exercise the informants should perform, as well as the directions to a specific gym. Option and volition has been related to adherence and degree of PA after ending an intervention (Edmunds, Ntoumanis, & Duda, 2008; Fortier, Sweet, O'Sullivan, & Williams, 2007; Ng et al., 2012), and environments offering various kinds of exercise increase feelings of choice and the potential for development (Huberty et al., 2013; Podlog & Dionigi, 2009). Many informants felt pressured to deliver results and blamed themselves when they failed. From a SDT stance, the specified details may have reduced feelings of choice, options, and joy, and consequently thwarted satisfaction of the psychological needs (Deci & Ryan, 2000; Hagger & Chatzisarantis, 2008). For some informants, this may have prevented more autonomous motivation from developing, particularly in cases when it resulted in stress, failure, and guilt, as this study found that those states negatively affects the assimilations process, hence motivation and long-term behavior change.

Changing ideas and one's self-image, and consequently increasing the stability in behavioral change (Patrick & Williams, 2012), is a gradual process that requires time, and a supportive and nurturing context (Deci & Ryan, 2000). Social networks are a contextual factor that has shown to be both emotionally and practically supportive (Huberty et al., 2013; Podlog & Dionigi, 2009). Research has also found that commitment to a group has positive influence on adherence, whereas duty and responsibilities toward family can be inhibiting (Podlog & Dionigi, 2009). The impact of others was reflected in the need for relatedness, support, and interaction, as well as competition and comparisons with others while exercising. It confirms the positive influences and stimulation that commitment to a group can have on adherence, and the importance of exercise in a socially permissive context with realistic role models (Huberty et al., 2013; Moore et al., 2011). Support from a partner and other significant others was emphasized as an important factor when prioritizing and allowing PA to be integrated into the informants’ lives. In accordance with earlier research (Huberty et al., 2013; Podlog & Dionigi, 2009), the informants had difficulties prioritizing themselves. Smaller, more tangible chores around the house, for the family, or at work, became visible and distinguishable. It resulted in positive rewards, while prioritizing exercise could cause feelings of guilt, with its above mentioned negative effect on the PA routines. Again, the results exemplify how the relational complexity that consists of expectations, responsibilities, needs, and emotional bonds influenced the informants’ participation and motivation. The described process of involving others, planning, and developing new values and interests in harmony with the exercise lifestyle further emphasizes the importance and complexity of integrating exercise into the greater part of the self and the identity (Patrick & Williams, 2012).

### Methodical restrictions

In order to increase trustworthiness of the study findings, the analytic procedure followed the Braun and Clarke (2006) 15-point checklist for thematic analysis. As such, it can be argued that this study provides trustworthy findings regarding individual experiences from taking part in a 6-month exercise intervention. The findings go beyond those of a traditional evaluation of an RCT, which previously has been viewed as the tradition and ideal to establish knowledge (APA, 2006; Biddle et al., 2001; Bohlin & Sager, 2011). Furthermore, as studies using a qualitative approach exploring individual experiences from taking part in exercise interventions is lacking, this study provides important insights to the body of knowledge. Nonetheless, this study is not without limitations. A recurring discussion in qualitative methods is the ability to step aside from ones prejudices and preconceptions (Braun & Clarke, 2006; Willig, 2013). Consequently, a limitation with the present study is the fact that the coding was done only by the first author, as such, the first author's preconceptions as a psychologist and familiarity with the area of research may have affected the results. However, the transcripts and the possible themes were continuously discussed within the research group. Furthermore, during data collection as well as analysis, the author actively brought prejudices, biases, and pre-understandings to consciousness by trying to intake an open and reflecting stance. Another limitation is the fact that some informants may have perceived the interviewer as involved in the stress-related research, possibly influencing their degree of social desirability.

In inductive studies, there is no claim to produce generalizable results (Willig, 2013). However, the individuals in the present study may be different, and supposedly have more resources, on issues such as initial motivation, openness to new things, and dedication to perform and produce results. The variation, or lack of, aspects like these are likely greater in the general population and in authentic contexts outside the realm of a controlled intervention, and consequently needs to be addressed. Furthermore, aspects such as the informants various backgrounds, that they initiated and ended the intervention at different times, and that some dropped out, might have impacted the result. These differences can be considered to represent the heterogeneity that naturally occurs in groups. However, studying homogenous groups could result in new discoveries, linked to aspects that affect the participation. Moreover, as previously mentioned, a majority of the participants were highly educated, which may have affected the results. The majority of psychological research, however, seems to be conducted with samples from western, educated, industrialized, rich, and democratic societies (Henrich, Heine, & Norenzayan, 2010). As such, we, as researchers, need to become better at recruiting more diverse samples (Henrich et al., 2010).

### Future practice and research

Psychological research has found that general factors are of great importance for treatment outcomes (APA, 2006; Bohlin & Sager, 2011). Supposedly, such factors can help the work with individuals’ motivation and persistence in behavioral change. One general factor of importance in the present study is the alliance (Norcross & Wampold, 2011). The results show that relational needs and expectations had an important part in the informants’ participation. As such, alliance building should be emphasized in future interventions. Using knowledge about individuals’ relational history and abilities, the coach's approach and support can be adjusted to fit the individuals’ specific needs. Some of the strategies that were used in the intervention to facilitate autonomous motivation were to foresee barriers and take an emphatic stance, which includes emphasizing and considering the individual's emotions and perspectives. Foreseeing external and internal barriers can be hard. In the present study, this was illustrated by the informants’ experiences, and the effects that the stress test had on their participation and exercise. The informants also experienced guilt, obligation, and feelings of disappointment and failure in relation to their participation and exercise. Consequently, a future challenge in exercise interventions is to work on and prepare for such aspects. A good alliance enables work on complex aspects of the self. It may be advisable to give a rational, to be observant, normalize, and reflect upon emotions associated with performance and prestige, and affect regarding the individuals’ bodies and physical abilities.

## Conclusion

The study shows that exercise involves and arouses often unpredictable emotional responses and thoughts about the individuals’ self, body, and relationships. To a varying extent the informants experienced that their specific needs were met during their participation. Also, the results show that their personal experiences, capacities, and life circumstances affected their motivation and to what extent they could adjust to the resources offered. Hence, it is important to maintain an individualized approach to standardized exercise interventions. Learning from psychological research, exercise interventions can be improved and autonomous motivation facilitated, by strengthening the alliance. Relational needs diverge between individuals. A good alliance establishes a foundation to work on the complex emotional, and consequently behavioral, aspects associated with PA. Autonomy supportive techniques, and long-term behavior change, can be improved by discussing individuals’ expectations, and normalizing and providing a rationale for emotions and other aspects that are difficult for individuals to predict and foresee.
